# A Hybrid Convolutional, Mamba and Spiking Neural Network Architecture Search for Seizure Prediction

**DOI:** 10.34133/cbsystems.0689

**Published:** 2026-09-30

**Authors:** Bo Yu, Chang Li, Rencheng Song, Xiang Liu, Ruobing Qian, Xun Chen

**Affiliations:** ^1^ Department of Biomedical Engineering, Hefei University of Technology, Hefei 230009, China.; ^2^ Anhui Province Key Laboratory of Measuring Theory and Precision Instrument, Hefei University of Technology, Hefei 230009, China.; ^3^ Department of Neurosurgery, the First Affiliated Hospital of USTC, Division of Life Sciences and Medicine, University of Science and Technology of China, Hefei 230001, China.; ^4^ Department of Electronic Engineering and Information Science, University of Science and Technology of China, Hefei 230026, China.

## Abstract

In recent years, deep learning technology has played an increasingly important role in seizure prediction based on electroencephalogram signals. However, the performance of deep learning algorithms is often highly dependent on the design of their neural network architectures; the process of manually designing neural network architectures is usually very time-consuming and resource-intensive. In addition, different types of networks have distinct advantages, and how to automatically hybridize these networks to design an effective network for seizure prediction remains a substantial challenge. To address this issue, this paper proposes a hybrid convolutional, Mamba, and spiking neural network architecture search framework (CMS-NAS) based on a multiobjective evolutionary algorithm for seizure prediction, which can automatically design a lightweight network capable of comprehensively extracting electroencephalogram features. By simulating the natural selection mechanism, CMS-NAS iteratively evolves candidate networks through populations and uses a multiobjective scoring function to simultaneously optimize the model’s prediction accuracy and model size. Extensive experiments are conducted on 2 public datasets and a private dataset, and the experimental results show that the network model searched by the CMS-NAS algorithm achieves competitive performance in seizure prediction.

## Introduction

Epilepsy is a common neurological disorder that seriously affects patients’ quality of life, with approximately 50 million people worldwide affected by it [1,2]. Electroencephalogram (EEG) can reflect the brain’s electrical activity in real time and plays a crucial role in the diagnosis and monitoring of epilepsy [3–5]. However, EEG signals inherently possess complex characteristics such as nonstationarity, high noise, and individual differences, which make manual interpretation of EEG signals not only time-consuming and labor-intensive but also prone to subjective biases [6,7]. Therefore, it is important to advance the development of automatic seizure prediction algorithms and explore efficient, reliable algorithms that can accurately classify epileptic signals.

Seizure prediction is essentially a pattern recognition task for multichannel time-series signals [8]. Early technologies relied on manual feature engineering combined with shallow classifiers such as support vector machine or *k*-nearest neighbor for discrimination [9,10]. With the evolution of artificial intelligence technology, the deep learning (DL) paradigm has substantially improved seizure prediction performance through end-to-end representation learning. For example, convolutional neural network (CNN) extract spatial features using local receptive fields; Thuwajit et al. [11] proposed a multiscale CNN that outperforms existing methods in seizure prediction, notably improving sensitivity and F1-score. However, the fixed receptive fields of CNN limit their global modeling capability. Long short-term memory (LSTM) network capture long-range dependencies in EEG through gating mechanisms; Tuncer and Bolat [12] used a Bi-LSTM network to classify EEG data, achieving an accuracy of 96% in distinguishing epileptic activities from other activities. Nevertheless, LSTM faces challenges such as difficulty in parallel computing and the risk of gradient vanishing. Transformers, equipped with unique attention mechanisms, exhibit excellent performance in global modeling; Hu et al. [13] proposed the Inresformer network, which combines residual networks and discrete wavelet transform to extract multiscale features of EEG, achieving extremely high accuracy on the Bonn dataset. However, the quadratic computational complexity of Transformers results in low efficiency in processing EEG signals. To address the limitations of Transformers, Gu and Dao [14] proposed the Mamba network, which enables global feature extraction with low computational complexity. In addition to the aforementioned artificial neural network, spiking neural network (SNN) greatly reduce network computation by simulating the mechanism of information transmission in biological neurons and have been widely applied in the field of EEG [15]. Compared to using the aforementioned technologies individually, the hybrid use of multiple technologies obviously has greater potential [16,17]. Shanmugam and Dharmar combined CNN and LSTM to develop a 1-dimensional (1D)–CNN–LSTM model, achieving extremely high accuracy in seizure prediction tasks [18]. Although some studies have hybridized these networks, how to integrate their respective advantages to design an energy-efficient network capable of effectively extracting EEG features remains a substantial challenge.

To address this issue, this work adopts a hybrid architecture combining CNN, Mamba, and SNN. Although these 3 networks have been separately utilized in the field of seizure prediction, there is still a lack of research on their integrated application to maximize their respective advantages. We conduct this study to fill such a research gap. We adopt lightweight convolutional networks equipped with depthwise separable convolutions to efficiently extract local time-frequency details from EEG signals. Mamba performs global long-range temporal modeling with near-linear computational complexity, which compensates for the limited capability of convolutional networks in capturing long-range dependencies. SNNs generate compact sparse features via sparse spike coding and further reduce the overall model size. The deep integration of the 3 components makes the architecture well suited for the inherent characteristics of EEG signals, including nonstationarity, high noise and long temporal ranges. However, when designing models that hybridize CNN, Mamba, and SNN, due to the enormous design space, manual network architecture design is time-consuming and resource-intensive, and it is difficult to balance network accuracy and efficiency. Therefore, there is a need for an effective algorithm that can automatically generate excellent network architectures, thereby reducing design costs. Among these methods, neural architecture search (NAS) has emerged as a popular research direction in recent years. NAS aims to automatically design high-performance networks by formulating neural network design as an optimization problem and searching for the optimal architecture through automated means. It typically takes validation accuracy as the primary performance metric, while other metrics such as computational efficiency may also be considered [19]. Existing methods cover categories such as reinforcement learning algorithm, gradient-based algorithm, and evolutionary algorithm [20]. Reinforcement learning methods formulate NAS as a markov decision process and obtain rewards based on the accuracy or efficiency of the generated networks [21]. Gradient-based NAS methods optimize architecture parameters via backpropagation through the continuous relaxation of discrete choices and reduce memory overhead and search time by restricting continuous activation paths or using stochastic gradient updates [22,23]. However, these methods usually focus on only one aspect of neural network performance, making it difficult to design neural networks that can optimize multiple aspects simultaneously. In contrast, evolutionary algorithms simulate natural selection mechanisms to iteratively evolve candidate networks through populations [24], making them suitable for multiobjective and discrete design challenges and more in line with the requirements of the seizure prediction field. Currently, there are limited studies on NAS in the field of seizure. Compared with NAS works in other research areas, this paper constructs a hybrid search space integrating CNN, Mamba, and SNN, which is tailored for seizure EEG prediction tasks. We also adopt a multiobjective weighted scoring function within the multiobjective evolutionary algorithm to realize the joint optimization of prediction accuracy and model lightweight design for seizure prediction.

Specifically, this paper proposes a hybrid convolutional, Mamba, and spiking neural network architecture search framework (CMS-NAS) based on a multiobjective evolutionary algorithm for seizure prediction. The search space of CMS-NAS incorporates 2 lightweight basic modules specially designed for EEG signal extraction. CMS-NAS completes population initialization by generating individual encodings via our custom-designed encoding scheme and automatically converts these encodings into corresponding concrete networks for training. Subsequently, it simultaneously evaluates the prediction accuracy and size of the models using a multiobjective weighted scoring function. Finally, by simulating the natural selection mechanism, it enables crossover and mutation operations among elite individuals to generate the next-generation population individuals with more possibilities. In this way, the prediction accuracy and size of the models are continuously optimized. CMS-NAS aims to find the Pareto optimal solution set of network architectures for seizure prediction from the vast search space encompassing CNN, Mamba, and SNN. The main contributions of this paper are as follows:

1. We propose an end-to-end CMS-NAS framework based on a multiobjective evolutionary algorithm to automatically search for the optimal hybrid Convolutional, Mamba, and SNN architecture for seizure prediction tasks.
2. To comprehensively balance prediction accuracy and resource efficiency, we propose a multiobjective evolutionary algorithm. We design a distinctive search space tailored for the seizure prediction task and adopt a multiobjective weighted scoring strategy, which simultaneously considers classification performance and model parameter quantity during the search process.
3. Extensive experiments are performed on 2 public datasets and the private dataset. The experimental results indicate that the proposed algorithm achieves competitive performance under identical experimental conditions, verifying its effectiveness.

## Methods

This section will elaborate on the CMS-NAS algorithm, whose workflow is illustrated in Fig. 1. The CMS-NAS algorithm is mainly composed of 2 parts, namely, the CMS-NAS search space and the search process.

**Fig. 1.**
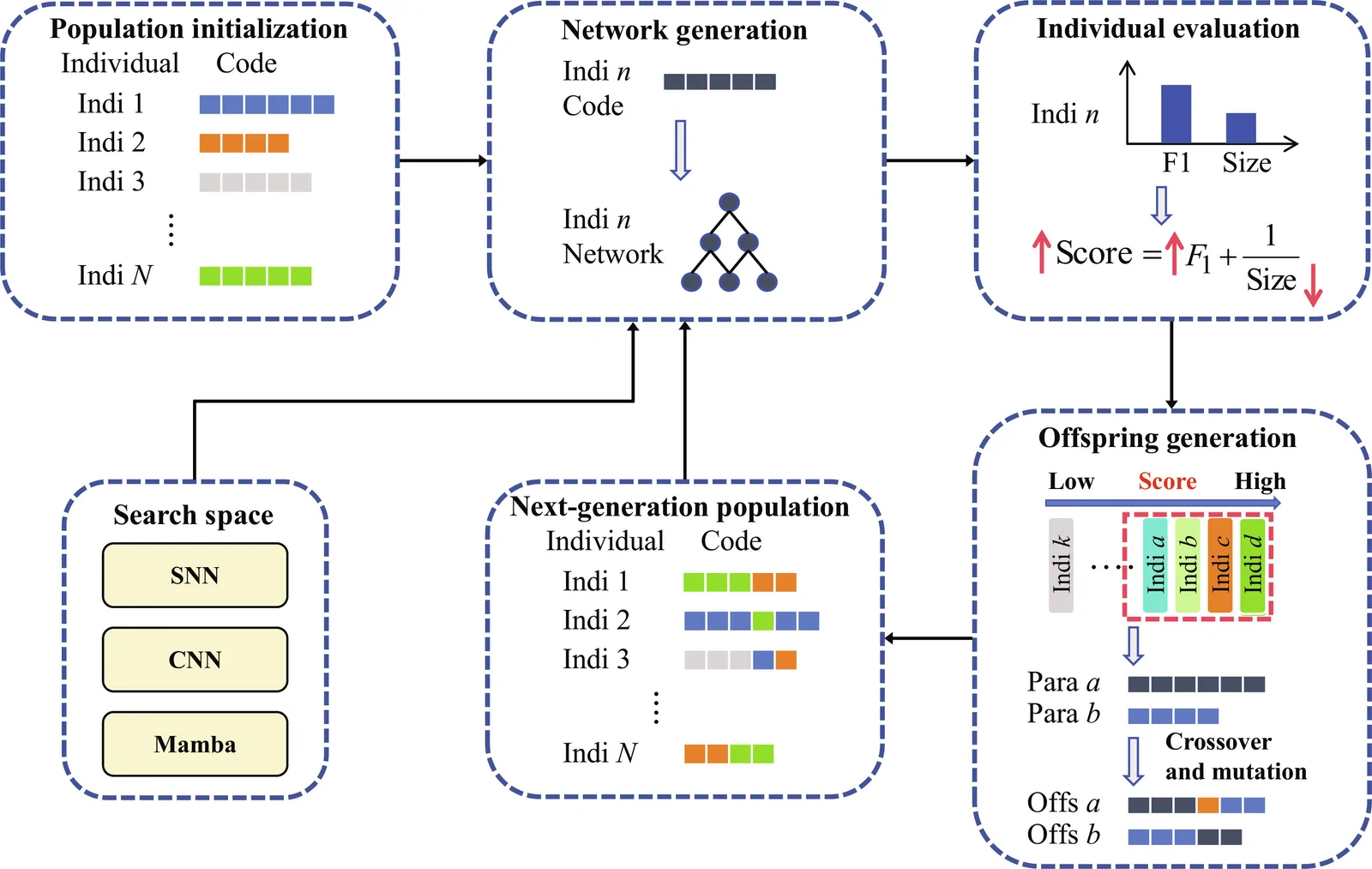
The main workflow of this paper.

### Search space of CMS-NAS

The search space of the CMS-NAS algorithm includes CNN, Mamba, and SNN, which are introduced separately below.

#### CNN

In the field of DL, CNNs are one of the most famous and widely used algorithms [25]. A CNN can automatically learn local feature representations of input data through mechanisms such as local receptive fields, weight sharing, and spatial downsampling. Essentially, it performs local weighted summation operations on input data through learnable convolution kernels. This method fully utilizes the structure of 2-dimensional input data such as image signals, requiring only a small number of parameters, simplifying the training process, and accelerating the network operation speed, which is consistent with the working mode of visual cortex cells [26]. Compared with other classification algorithms, CNN requires fewer preprocessing operations. In traditional methods, filters need to be designed manually, while CNN can automatically learn these features through sufficient training. CNN has been widely applied in various fields such as computer vision, speech processing, and face recognition [27].

In order to extract local features from EEG signals with a low parameter count, depthwise separable convolution is adopted in this paper. Unlike standard convolution that performs spatial convolution and channel mixing simultaneously, depthwise separable convolution first uses depthwise convolution to capture local features within each channel, while pointwise convolution further fuses information across different channels, thereby substantially reducing the parameter count and computational complexity. This design is suitable for seizure prediction tasks with limited sample size, large interpatient variability, and the requirement for lightweight models.

#### Mamba

In recent years, a novel architecture Mamba inspired by classic state space model (SSMs) has emerged as a potential alternative for building foundation models. While maintaining modeling capabilities comparable to Transformers, it achieves near-linear scalability with respect to sequence length [28]. As shown in Fig. 2, the Mamba module adopted in this study mainly consists of 4 key components: a positional encoding layer, input and output projection layers, 1D causal convolution, a SSM layer, and a gating mechanism. It realizes content-adaptive long-sequence modeling by dynamically generating SSM parameters, which can effectively extract global features in signals.

**Fig. 2.**
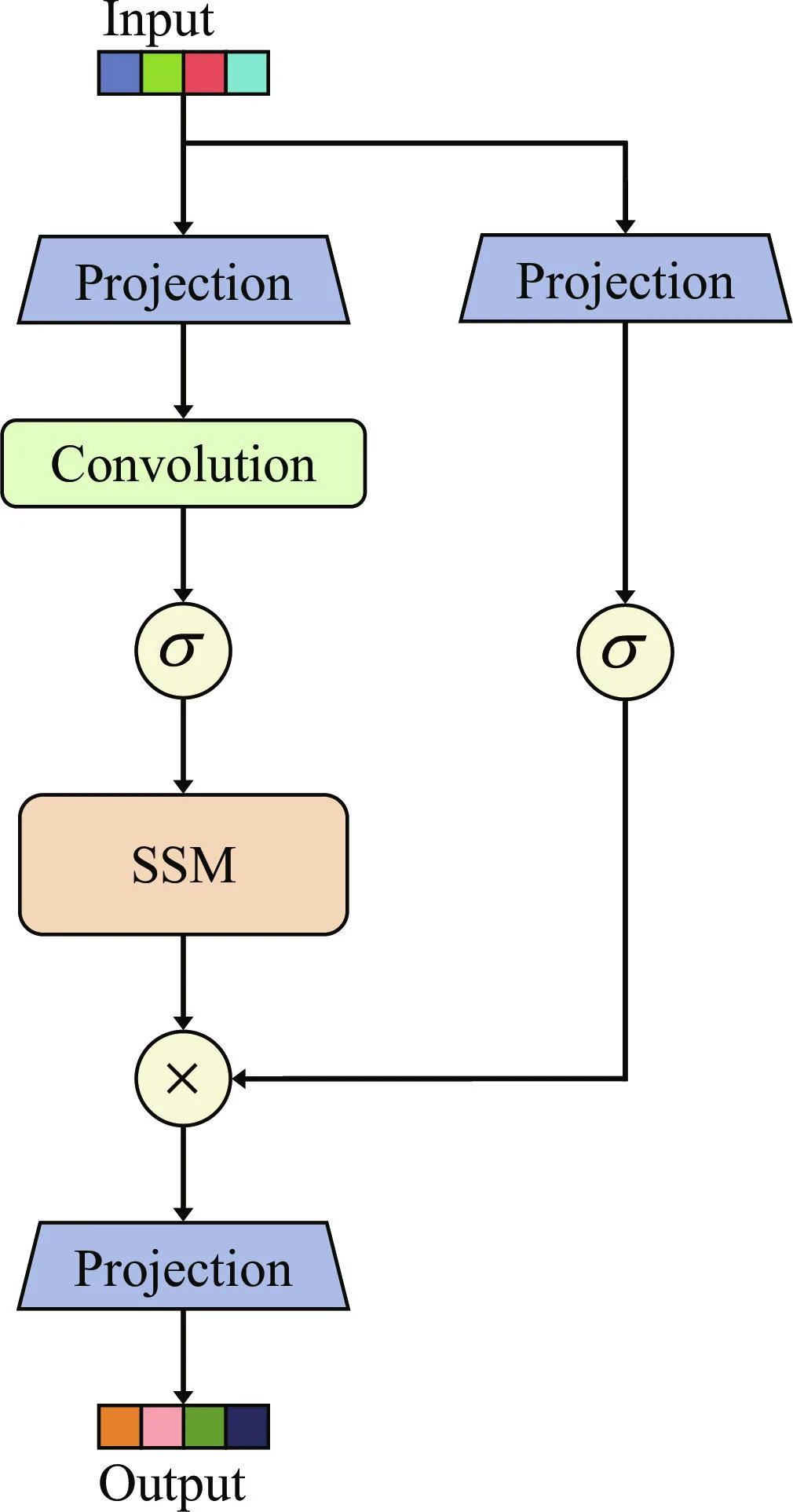
Structure of the Mamba module.

The Mamba module is built based on the SSM, adjusting the state evolution path through input-dependent dynamic parameters. The SSM is modeled through the following state update and output equations:

$$\left\{\begin{array}{cc} h\left(t\right)=A\left(t\right)\cdot h\left(t-1\right)+B\left(t\right)\cdot x\left(t\right) & \\ y\left(t\right)=C\left(t\right)\cdot h\left(t\right) & \end{array}\right.$$ (1)

where *x*(*t*) denotes the input EEG signal; *h*(*t*) denotes the hidden state vector; *A*(*t*), *B*(*t*), and *C*(*t*) are the state transition, input projection, and output projection matrices, respectively, and *y*(*t*) is the output signal. Mamba enables the network to autonomously focus on key rhythmic components in EEG time-frequency features, balancing training parallelism and inference efficiency.

#### SNN

As a third-generation neural computing model, SNN encode information in the form of discrete spike sequences by simulating neuronal spike firing behavior. Relying on their event-driven and sparse computation biomorphic characteristics, SNN can ssubstantially reduce network energy consumption and naturally align with the temporal nature of EEG signals [29,30]. Spiking neurons are the basic units of SNN and have strong biological interpretability. Among them, the leaky integrate-and-fire (LIF) neuron model is widely used, which achieves a balance between simplicity and bioinspired characteristics [31]. The membrane potential dynamics of the neuron follow:

$$u\left(t_{c}\right)=-\frac{1}{\tau }u\left(t_{c}\right)+\frac{w}{\tau }x\left(t_{c}\right)$$ (2)

where τ∈ℝ is the membrane time constant, *u*(*t*) denotes the membrane potential of the neuron at time *t*; *w* is the learnable weight, and $x\left(t\right)$ is the input at time *t*. By comparing Eqs. (1) and (2), we can find that both Mamba and LIF follow the first-order recursive paradigm summarized as “decay of historical states + weighted accumulation of current inputs = output”. Mamba realizes global context modeling of EEG signals and resolves the challenge of long-range dependencies. The LIF neuron maps dense real-valued features to binary sparse spike trains, so as to eliminate redundant information and reduce feature dimensionality. The joint use of the 2 components can fully exploit this recursive feature extraction mechanism.

Neurons emit spikes using a step function:

$$S\left(t\right)=\left\{\begin{array}{cc} 1, & \text{if }u\left(t\right)\geq V_{\mathrm{th}}\\ 0, & \text{otherwise} \end{array}\right.$$ (3)

where *S*(*t*) is the spike output of the LIF neuron, and $V_{\mathrm{th}}\in \mathbb{R}$ is the membrane potential threshold. Specifically, when the membrane potential *u*(*t*) exceeds the threshold $V_{\mathrm{th}}$, a spike is generated; when the membrane potential is below the threshold, no spike is generated. After generating a spike, the membrane potential will become the reset potential $V_{\mathrm{res}}\in \mathbb{R}$. To enable gradient backpropagation, this paper adopts the zero-order forward function. During forward propagation, the zero-order forward function performs hard-threshold spike generation as specified in Eq. (3). During backpropagation, a triangular window is used to approximate the gradient near the threshold, and the formula is as follows:

$$\mathrm{Sg}=\text{max}\left(\gamma -|\;u\left(t\right)-V_{\mathrm{th}}|\;,\;0\right)/\gamma^{2}$$ (4)

where Sg denotes the surrogate gradient value, and γ controls the width of the triangular surrogate gradient window and is set to 1. This effectively addresses the nondifferentiable problem in the spike generation process.

#### Two basic modules

As shown in Fig. 3, in the search space of this paper, CNN, Mamba, and SNN are not simply cascaded as 3 independent networks but encapsulated into 2 hybrid modules, namely, the ConvSpike module and the MambaSpike module, for subsequent architecture search.

**Fig. 3.**
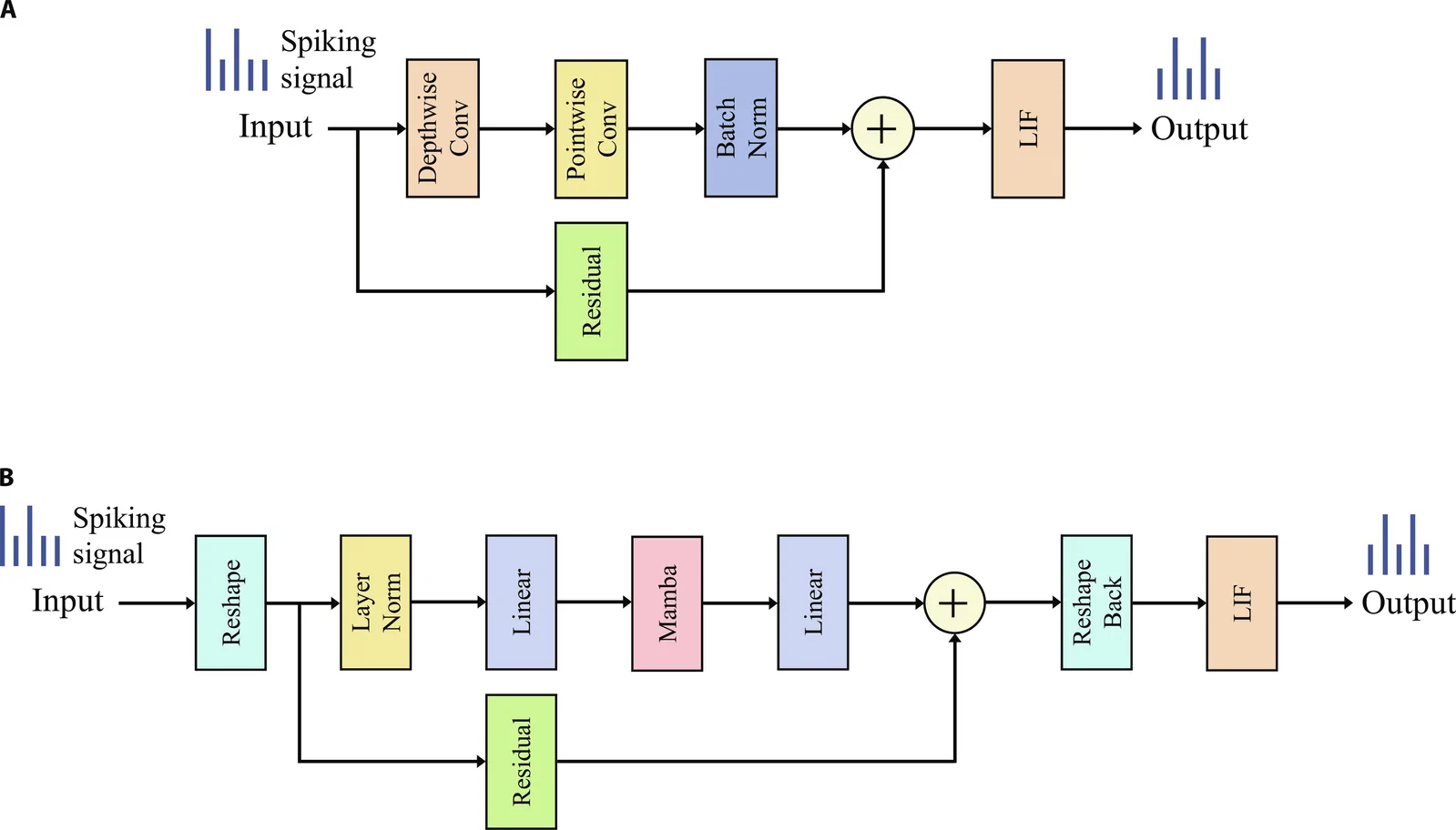
Panel (A) illustrates the architecture of the ConvSpike module, and panel (B) illustrates the structure of the MambaSpike module. LIF, leaky integrate-and-fire.

The original EEG signal is a multichannel time series, denoted as $X\in {\mathbb{R}}^{C\times L}$, where *C* is the number of lead channels and *L* is the length of discrete sampling points in the time dimension. Before being fed into the model, the short-time Fourier transform (STFT) is first adopted to convert each channel signal into a time-frequency representation $X\in {\mathbb{R}}^{C\times F\times W}$, where *F* denotes the frequency resolution and *W* represents the number of time frames. EEG is a typical nonstationary signal, and the discriminative patterns prior to epileptic seizures are usually reflected in time-varying spectral changes rather than merely the original waveform morphology. Meanwhile, to better exploit the characteristics of SNN, the STFT-processed time-frequency data $X\in {\mathbb{R}}^{C\times F\times W}$ is divided into *T* time-step data $X\in {\mathbb{R}}^{T\times C\times F\times \frac{W}{T}}$ according to the number of time frames. With the introduction of time steps, the model is no longer an ordinary “STFT image classifier”, but a time-frequency dynamic model with an internal temporal structure. Each time step is treated as an EEG event, enabling LIF neurons to perform cross-time-step membrane potential accumulation and spike firing, which fully leverages the bioinspired characteristics of SNN including event-driven computation and sparse temporal gating mechanisms.

The data $X\in {\mathbb{R}}^{T\times C\times F\times \frac{W}{T}}$ is fed into the ConvSpike module. The ConvSpike module employs depthwise separable convolution to extract local patterns and perform channel transformation on 2D STFT time-frequency maps. Subsequently, learnable LIF neurons perform membrane potential accumulation and threshold firing along the temporal step dimension, converting continuous convolutional features into spike-based features and thus introducing a sparse temporal gating mechanism. It combines the local feature extraction capability of CNN and the sparse, low-computation characteristics of SNN, enabling lightweight extraction of EEG features. The MambaSpike module first performs weighted summation along the frequency dimension to compress and reconstruct the features. Subsequently, it employs Mamba for feature modeling to implement global context modeling of EEG signals. After reshaping the features, the LIF neurons map the dense real-valued features into binary sparse spike sequences, which eliminates redundant information and compresses the feature dimensions. This design integrates the global modeling capability of Mamba and the sparse, low-consumption computing characteristics of SNN. This hybrid design allows the model to focus on local features and global temporal features at the same time. It removes the additional computational burden incurred when sequence models are directly used to process complete time-frequency maps. Furthermore, the spike-based mechanism boosts the model’s sensitivity to transient abnormal responses while maintaining a low parameter count.

### Search process of CMS-NAS

The CMS-NAS framework adopts a classic evolutionary paradigm to realize automated architecture exploration, following an iterative evolutionary cycle, as shown in Algorithm 1. Firstly, an initial population is generated through a random encoding strategy, enabling candidate architectures to have a wide distribution in terms of topology, depth, and width (Line 1). Subsequently, each individual is comprehensively evaluated based on a multiobjective weighted scoring mechanism to obtain its actual performance in the seizure prediction task (Line 3). Furthermore, based on the multiobjective weighted scoring results, crossover, mutation, and environmental selection operations are performed on the elite individuals in $P_{g}$ to generate the next-generation population $P_{g+1}$ (Line 4). This process iterates continuously until the preset maximum number of evolutionary generations is reached and finally outputs the Pareto optimal solution set, providing decision-makers with a series of candidate architectures that balance between model accuracy and the number of model parameters.


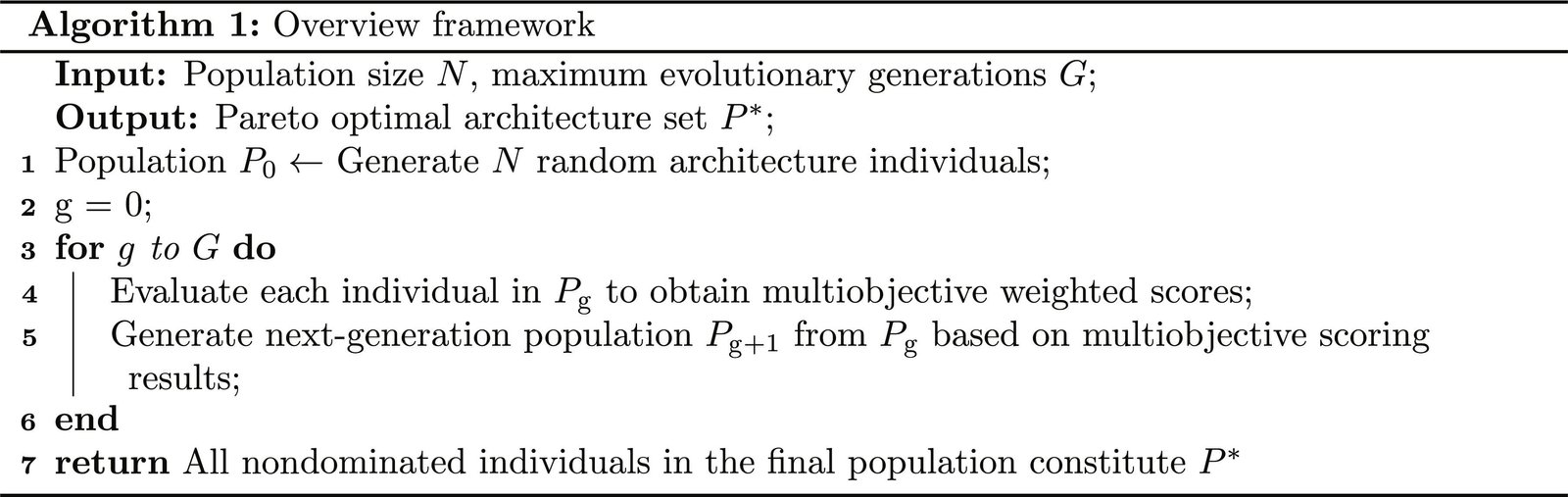


#### Population initialization

Population initialization is the starting point of evolutionary search, and its quality directly affects the optimization efficiency of subsequent optimization. To ensure that the initial population can fully cover the entire architectural search space, this paper designs an encoding scheme and adopts a hierarchical random strategy. Algorithm 2 describes the generation procedure of the initial population. Specifically, the encoding generation of each individual first confirms the time step *T* adopted by the corresponding individual (Line 2). Afterward, the network depth is assigned with an integer randomly sampled within the predefined interval (Line 3). Then, each basic module is encoded sequentially: The automatic algorithm first judges whether the basic module belongs to the ConvSpike module or the MambaSpike module (Line 5) and finally determines the searchable parameters of the target module. The configurable parameters of the ConvSpike module include output channel number, kernel size, and stride; those of the MambaSpike module cover output channel number and the feature dimension of Mamba (Line 6). All individuals are encoded following the above pipeline to finish population initialization.


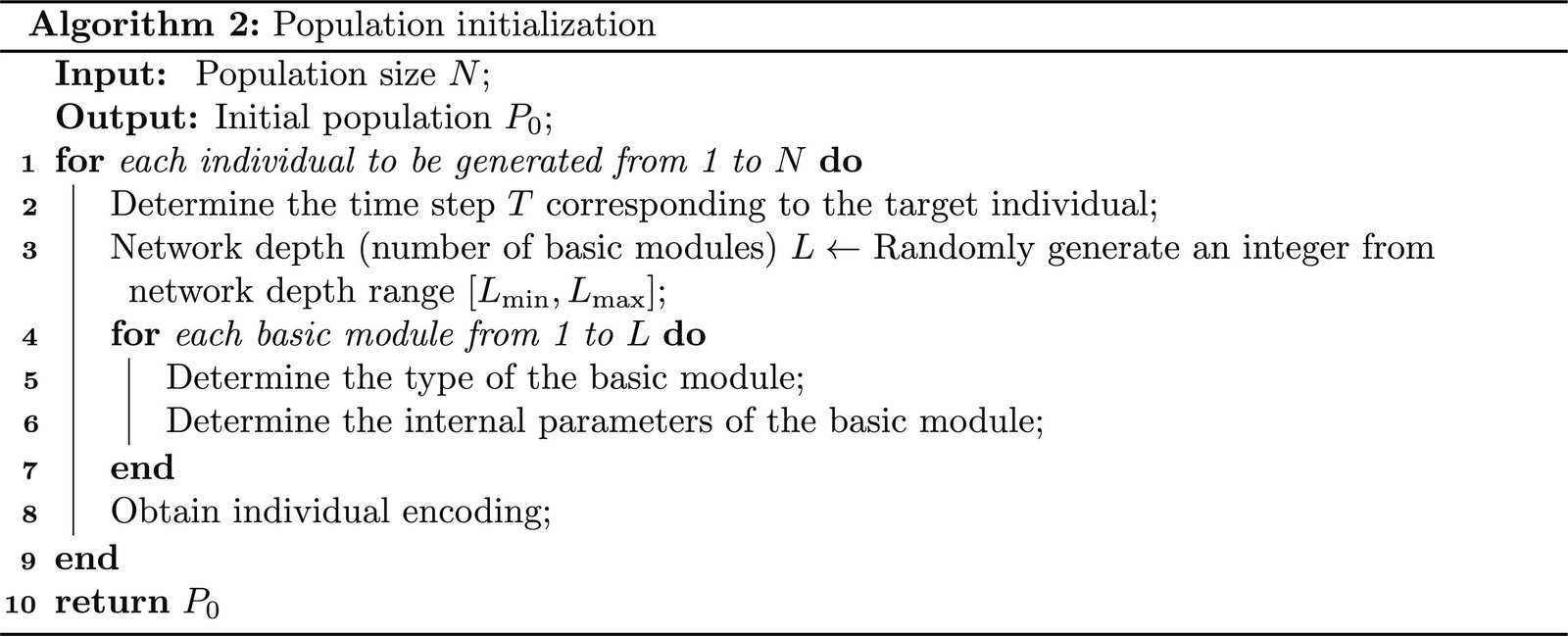


To better understand this encoding strategy, Fig. 4 presents a specific individual encoding and its corresponding network structure. As shown in Fig. 4A, this individual has a time step of 2 and a network depth of 3, i.e., consisting of 3 basic modules. The first basic module is a ConvSpike module. Its number of input channels is fixed to the channel count of epileptic EEG data, while the number of output channels is set to 64 as generated by the algorithm. The kernel size and stride are also illustrated in Fig. 4A. The second basic module is a MambaSpike module, which connects the output of the first module. Its number of input channels defaults to 64, and the parameter settings related to the Mamba model and the number of output channels are also presented in Fig. 4A. The third basic module is also a ConvSpike module. However, since the frequency dimension has been compressed after the MambaSpike module, a 1D convolutional kernel is adopted instead. Fig. 4B depicts the detailed network structure corresponding to this encoding. The network employs 3 basic modules, followed by a global pooling layer and a multilayer perceptron layer to complete the classification task.

**Fig. 4.**
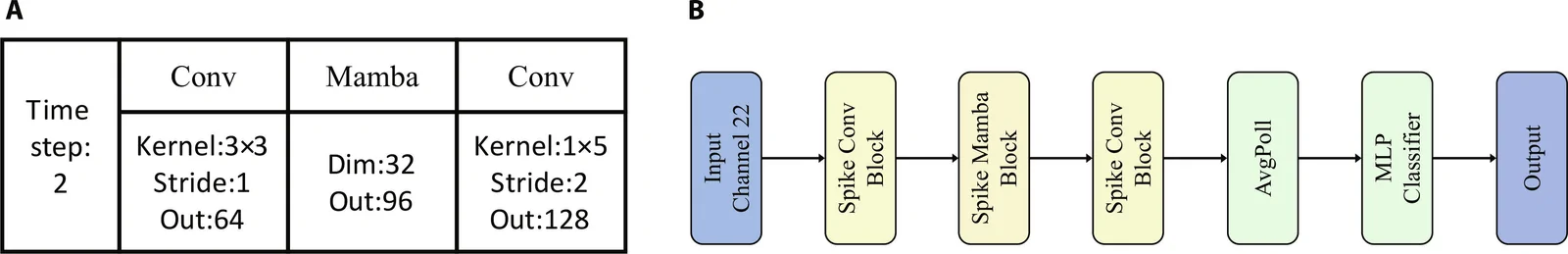
Panel (A) shows a concrete individual encoding, and panel (B) presents the network structure corresponding to this encoding.

#### Individual evaluation

Individual evaluation is the most computationally intensive step in the framework, and its accuracy directly determines the effectiveness of the search direction. We propose a multiobjective scoring function that normalizes and weighted aggregates the F1-score of the model’s prediction results and the number of model parameters, which not only retains the trade-off characteristics of multiobjective optimization but also reduces the complexity of the selection operation. This design ensures that the algorithm efficiently converges to the high-quality solution region within a limited number of generations.

Algorithm 3 describes the individual evaluation process. The evaluation process starts with model instantiation, where the system generates the corresponding model structure according to the individual encoding (Line 1). The model is then trained using the training dataset (Line 2). Then, the number of model parameters $P_{\mathrm{num}}$ is automatically calculated by the algorithm (Line 3). After each training epoch, the model calculates the classification F1-score on the validation set and records the historical maximum value as the prediction performance indicator $F1_{\text{best}}$ of the architecture (Lines 4 to 8). Finally, the final score of each individual is obtained through the multiobjective scoring function (Line 9).


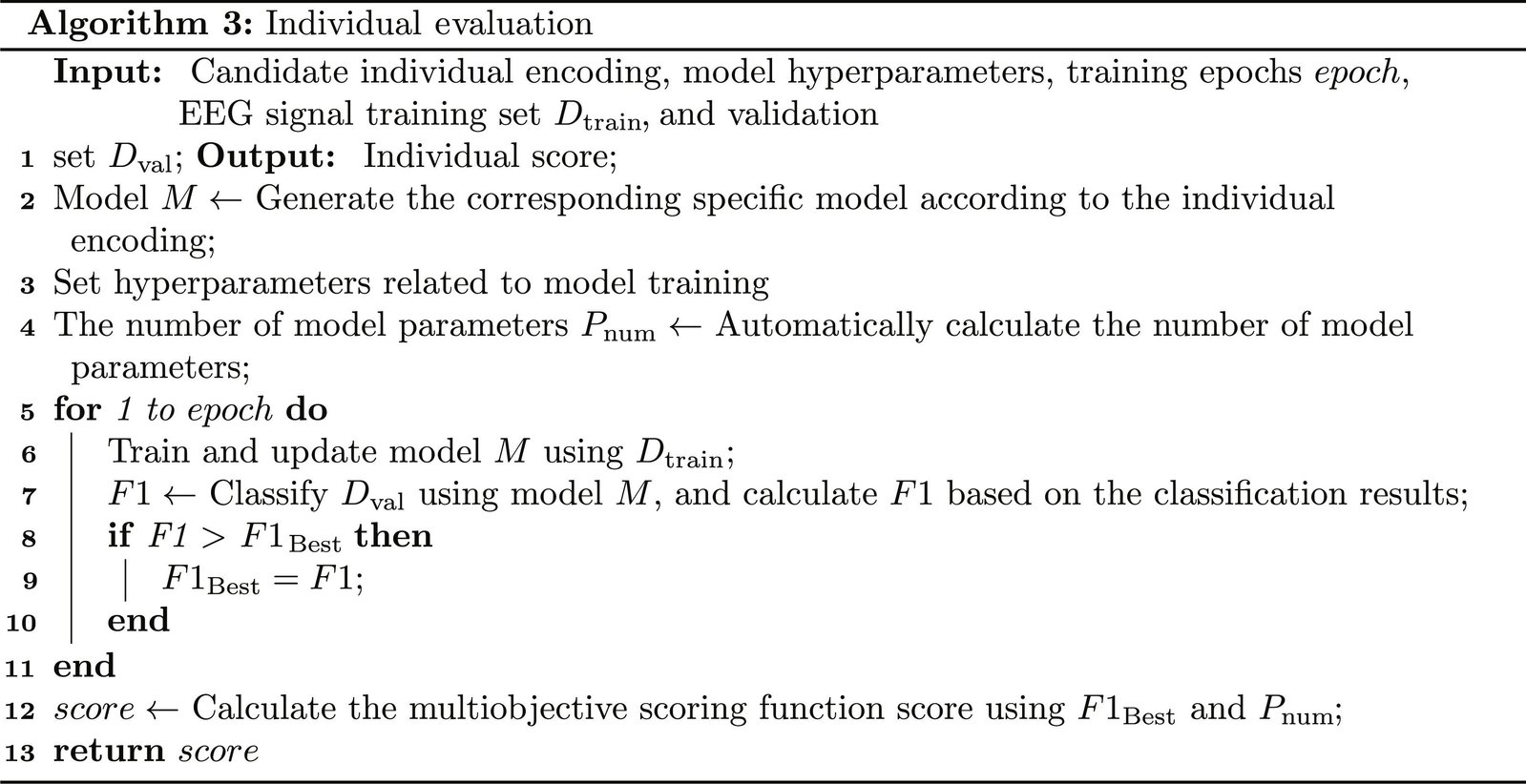


To comprehensively measure architecture efficiency, the algorithm adopts 2 conflicting objectives: model classification accuracy and the number of model parameters. Specifically, the F1-score of the model’s validation results is used to measure model classification accuracy. The F1-score can fully reflect the model’s ability to rank and classify positive and negative samples in EEG signals, and in classification tasks with imbalanced sample classes such as epileptic EEG signals, a higher F1-score indicates better robustness of the model. The number of model parameters directly reflects the model’s latency, power consumption, and the difficulty of deploying it on real-time brain–computer interfaces or embedded devices; a smaller model is more in line with the requirements for seizure prediction. Higher F1-score and lower number of model parameters are conflicting in the scoring process. Therefore, before scoring, $F1_{\text{loss}}$ is obtained through 1−F1 as the inverse accuracy of the model. In this way, the smaller the inverse accuracy (the better) and the smaller number of model parameters (the better) are in the same direction during scoring, facilitating multiobjective weighted aggregation.

The $F1_{\text{loss}}$ value is between 0 and 1, while the order of magnitude of the number of model parameters is usually $10^{6}$. To make the 2 objectives with substantially different orders of magnitude comparable, we first normalize each indicator through Eq. (5), compressing it to the interval [*D*, 1.0] to avoid extreme values dominating the optimization:

$${\text{norm}}_{F1}\left(x\right)=D+\left(1-D\right)\times \frac{F1_{\text{loss}}\left(x\right)-F1_{\min}}{F1_{\max}-F1_{\min}}$$ (5)

where ${\mathrm{norm}}_{F1}\left(x\right)$ is the normalized $F1_{\text{loss}}$ metric, *D* is the lower bound of the normalization interval, $F1_{\text{loss}}\left(x\right)$ is the $F1_{\text{loss}}$ value of the current individual, and $F1_{\min}$ and $F1_{\max}$ represent the minimum and maximum values of $F1_{\text{loss}}$ among all individuals in the current population, respectively. The normalization of model size is similar. Subsequently, a multiobjective scoring function is introduced to convert multiobjective scores into a single fitness score:

$$\text{Score}\left(x\right)=\lambda_{\mathrm{acc}}\times \frac{1}{{\text{norm}}_{F1}\left(x\right)}+\lambda_{\text{size}}\times \frac{1}{{\text{norm}}_{\text{size}}\left(x\right)}$$ (6)

where $\lambda_{\mathrm{acc}}$ is the model accuracy weight and $\lambda_{\text{size}}$ is the number of model parameters weight. By setting different weights, the algorithm can focus on different evolutionary directions. This design not only retains the trade-off characteristics of multiobjective optimization but also simplifies the selection operation, enabling the algorithm to efficiently identify high-quality architectures that balance error, energy consumption, and storage. It should be emphasized that the test set is completely isolated during the entire architecture search process, including population evolution, individual evaluation, hyperparameter tuning, and early stopping.

#### Next-generation population

The generation of offspring populations and environmental selection are the core driving forces of evolutionary search, and their design directly determines the convergence speed and solution set quality of the algorithm. This framework adopts a hybrid strategy combining elitism preservation and diversity maintenance to ensure the continuation of high-quality genes while avoiding premature convergence to local optima.

Algorithm 4 describes the process of generating the next-generation population. At the beginning of each generation of evolution, first, based on the multiobjective scoring function evaluation results of the population individuals, the top 50% of individuals with the highest scores are selected to form the parent population (Line 1). This selection mechanism concentrates computing resources on exploring high-quality regions, improving search efficiency. To avoid homogenization of individuals in the elite pool, a random pairing strategy is adopted to generate parent combinations, which not only ensures that high-scoring individuals participate in reproduction but also prevents the loss of genetic diversity caused by inbreeding.


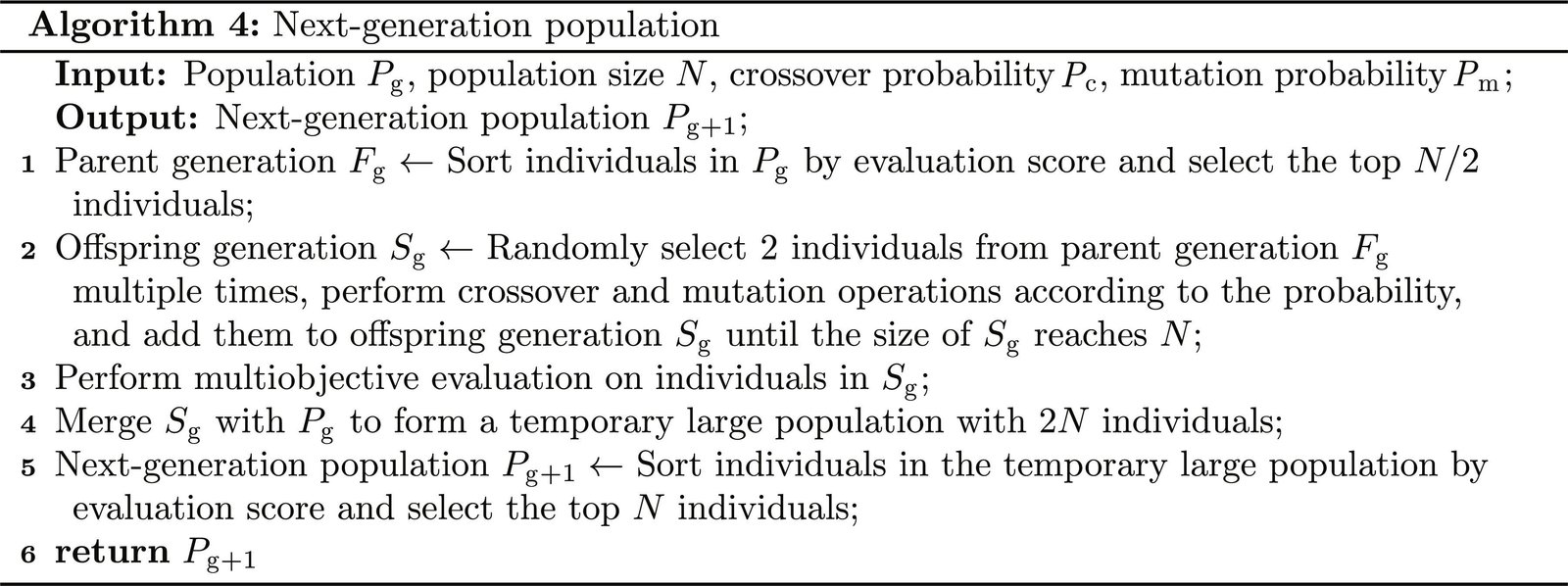


The crossover operation simulates the biological gene recombination process. For each pair of parents, a split position is randomly selected and the subsequent structure is swapped to generate 2 offspring (Line 2). This strategy realizes the transfer and fusion of architectural characteristics by recombining excellent modules of different individuals. The mutation operation applies local perturbations to individuals with a low probability. Such random exploration mechanisms help escape local optima and discover novel structural patterns. To prevent the generation of invalid networks, the algorithm automatically predicts structural legality. After crossover and mutation, the input and output channels of the affected units need to be recalculated to ensure the coherence of the computation graph.

After the generation of offspring, multiobjective scoring is performed on the individuals in the offspring population (Line 3). Then, environmental selection is executed to determine the next-generation population. This process merges the parent population $P_{g}$ and the offspring population to form a temporary population (Line 4), reorders them according to the multiobjective scoring results of the individuals, and truncates to the original size *N* (Line 5). This elitism preservation strategy ensures that the optimal individuals of all generations are not eliminated, realizing performance improvement. At the same time, since the offspring are generated by crossover and mutation, their diversity is injected into the new generation, maintaining the evolutionary ability of the population. This mechanism effectively drives the population to continuously approach the Pareto frontier under the condition of limited computational resources.

## Results

### Experimental data and preprocessing

In this study, model performance is evaluated on the Children’s Hospital Boston Massachusetts Institute of Technology Scalp EEG Database (CHB-MIT), the American Epilepsy Society Seizure Prediction Challenge Dataset (AES), and a private dataset [32,33]. As shown in Table 1, this experiment adopts continuous scalp EEG signals from 18 patients in the CHB-MIT dataset, with a total duration of 340.2 h. Each patient has experienced more than 2 epileptic seizure events, totaling 91 seizure events. The signals are recorded using the International 10–20 Electrode Placement System with a sampling rate of 256 Hz. The AES dataset uses continuous intracranial EEG signals from 4 dogs, with a total duration of 532 h and 38 seizure events, recorded at a sampling rate of 400 Hz.

**Table 1.** A summary of the CHB-MIT, AES, and private datasets

Dataset	Subject	Interictal hours	Seizures	Sample number	Sampling rate	Electrodes
CHB-MIT	18 Patients	340	91	493,818	256	22
AES	4 Dogs	532	38	745,081	400	16
Private	20 Patients	741	257	1,038,506	500	22

CHB-MIT, Children’s Hospital Boston Massachusetts Institute of Technology Scalp EEG Database; AES, American Epilepsy Society Seizure Prediction Challenge Dataset

The private dataset consists of continuous EEG recordings of seizure patients collected in real clinical hospital settings [34]. The data are annotated and maintained by a professional clinical team with decades of neurosurgical experience. Recordings follow the International 10–20 electrode placement system at a sampling rate of 500 Hz, with 22 channels acquired via bipolar derivation. The dataset includes 20 patients aged 8 to 60 years. The monitoring duration per patient ranges from 8 to 60 h, with a total EEG recording duration exceeding 700 h. Each patient experienced at least 3 epileptic seizure events during recording. The study protocol was reviewed and approved by the Ethics Committee of the First Affiliated Hospital of University of Science and Technology of China (approval No. 2025-RE-036, in accordance with the Measures for the Ethical Review of Life-Science and Medical Research Involving Humans issued by China in 2023).

Essentially, seizure prediction requires distinguishing between interictal and preictal phases in EEG signals. However, 3 datasets have substantial class imbalance issues, where interictal data of subjects is usually much more than preictal data. In most studies, the window length ranges from 5 to 30 s. In this experiment, a sliding window method is used to segment EEG signals from the 3 datasets into 5-s segments, which serve as samples for neural network training, validation, and testing. To balance the number of 2 types of samples during training, the overlapping oversampling technique is applied to the preictal data of subjects, and the focal loss function is adopted in the training process. This enables model training to focus on hard-to-classify samples and avoids the dominance of simple samples from the majority class over model training [35].

### Implementation details

During the search process of the CMS-NAS algorithm, the experiment adopts the settings and methods shown in Table 2. The total size of the search space can be derived from the combinatorial counting of global hyperparameters and module-level parameters, as formulated below:

$$S_{\text{total}}=N_{T}\times \sum_{L=L_{\min}}^{L_{\max}}N_{\text{module}}^{L}$$ (7)

where *N_T_* = 5 is the number of optional global time steps, $L_{\min}=2$ and $L_{\max}=10$ are the bounds of network depth, and $N_{\text{module}}=45$ is the total configurations per module position (5 × 3 × 2 for ConvSpike, 5 × 3 for MambaSpike). Through the setting of these parameters, the search space of the algorithm reaches the order of 1.74 × 10^17^, providing a foundation for the algorithm to effectively generate high-performance models. Meanwhile, by adjusting $\lambda_{\mathrm{acc}}$ and $\lambda_{\text{size}}$, the algorithm focuses more on the classification ability of the model during the search process.

**Table 2.** Key parameter settings of CMS-NAS

Parameter category	Parameter name	Parameter value
Population encoding evolution	Population iteration count *G*	20
	Population size *N*	20
	Time step	{2, 3, 4, 5, 6}
	Network depth range	[2, 10]
	Basic block channel	{64, 72, 84, 96, 128}
	Dimension of Mamba	{32, 64, 80}
	ConvSpike module kernel size	{1, 3, 5}
	ConvSpike module kernel stride	{1, 2}
	Crossover probability *P*_c_	0.9
	Mutation probability *P*_m_	0.7
Model training and evaluation	Learning rate	0.001
	Loss function	Focal loss
	Optimizer	AdamW;
		Weight decay = 0.0001
	Learning rate scheduler	Cosine annealing
	Training epochs	30
Multiobjective scoring function	Normalization range	[0.5, 1]
	Accuracy weight *λ*_acc_	1
	Model parameters weight *λ*_size_	1

CMS-NAS, convolutional, Mamba, and spiking neural network architecture search framework

When evaluating the prediction and generalization capabilities of high-quality models searched by the CMS-NAS algorithm, for the patient-specific setting: This experiment adopts the leave-one-seizure-out cross-validation method. If a patient has *M* seizure events, the EEG signals of 2 distinct states are divided into *M* chronological segments. In each round, the segment corresponding to 1 seizure event is selected as the test set to calculate evaluation metrics, and the remaining segments are used for model training and validation. For the patient-independent setting: This experiment adopts the leave-one-patient-out cross-validation method. Assuming the dataset contains *N* patients, the data of each patient are separately taken as the test set in sequence, while the data of the rest patients serve as the training set. This procedure repeats *N* times. For both settings above, the average value of evaluation metrics across all patients is taken as the final experimental result [35].

In evaluating the model’s epileptic seizure prediction capability, event-based metrics widely used in recent studies are adopted, including the area under the curve (AUC), sensitivity ($S_{n}$), false-positive rate (FPR), and statistical significance metric (*p*) [36,37]. The experiments adopt the *k*-of-*n* strategy, i.e., an alarm is issued only if at least *k* out of *n* consecutive predictions are positive. In this study, a positive event is defined when at least 20 out of 30 EEG signal samples are predicted as positive. A positive event predicted within preictal segments is counted as 1 true-positive event, while a positive event occurring during interictal segments is counted as 1 false-positive alarm event. $S_{n}$ is calculated as the proportion of successfully predicted true-positive alarms relative to the total number of seizure events. The FPR in this paper strictly denotes the number of false-positive alarm events per hour of interictal EEG recording, which corresponds to the clinically concerned hourly false alert count. Furthermore, across all experiments, the prediction horizon is uniformly set to 1 min and the seizure occurrence period to 0.5 h, so as to improve the clinical effectiveness of seizure prediction.

For subject-level *P* value calculation, we adopt a 1-tailed Poisson-binomial test with a Poisson random predictor sharing the identical subject-specific FPR as the comparison baseline. Each subject serves as an independent reporting unit, and each seizure event is treated as a trial unit, with all statistics computed based on leave-one-seizure-out cross-validation results. The probability that the random predictor generates at least one valid alert within the 0.5-h seizure occurrence period window is defined as $q=1-\exp\left(-\mathrm{FPR}\cdot \mathrm{SOP}\right)$. The 1-tailed *P* value corresponds to the upper-tail binomial probability that the Poisson-process baseline achieves no fewer than *n* successful predictions among total *N* seizures under the null hypothesis, formulated as:

$$P=\sum_{i=n}^{N}\left(\begin{array}{c} N\\ i \end{array}\right)q^{i}{\left(1-q\right)}^{N-i}$$ (8)

If P<0.05, we reject the null hypothesis and conclude the model outperforms the Poisson-process baseline.

### Performance comparison

Extensive comparative experiments are implemented under identical experimental conditions against multiple baseline algorithms, consisting of artificially architected models including spatio-temporal-spectral hierarchical graph convolutional network (STS-HGCN) [38], Vision Transformer (ViT) [39], hybrid vision transformer with data uncertainty learning (HViT-DUL) [40] and Long Short-Term Memory-Transformer and Self-Supervised Learning (SALT) [41], as well as automatically searched NAS models including Progressive Evaluation and sub-Population preservation Neural Architecture Search (PEP-NAS) [42], Lightweight Multiscale neural architecture search with Spectral-Spatial attention (LMSS-NAS) [43], Neural Architecture Search for Binary Neural Networks (BNN-NAS) [44] and efficient and lightweight convolutional neural network architecture search (SL-NAS) [45]. The seizure prediction performance on 3 datasets is summarized in Tables 3 to 5.

**Table 3.** Experimental results on the CHB-MIT dataset. All experiments are implemented under identical experimental settings, and each experiment is repeated more than twice. The optimal value of each evaluation metric is marked in bold.

Architecture design	Method	Patient-specific				Patient-independent		
		AUC	*S_n_* /%	FPR	*P* < 0.05	AUC	*S_n_* /%	FPR
Artificial design	STS-HGCN	0.869 ± 0.024	85.4 ± 5.6	0.155 ± 0.041	15/18	0.597± 0.027	42.7 ± 3.3	0.341 ± 0.026
	ViT	0.852 ± 0.031	82.3 ± 6.2	0.211 ± 0.052	15/18	0.612± 0.013	53.6 ± 3.0	0.304 ± 0.031
	HViT	0.922 ± 0.020	87.5 ± 4.9	0.146 ± 0.048	16/18	0.638± 0.020	58.2 ± 1.5	**0.206** ± **0.018**
	SALT	0.844 ± 0.032	83.3 ± 6.4	0.173 ± 0.047	14/18	0.604± 0.014	49.1 ± 2.7	0.368 ± 0.025
Automated design	SL-NAS	0.817 ± 0.035	86.5 ± 6.5	0.228 ± 0.055	13/18	0.614± 0.026	47.5 ± 2.4	0.312 ± 0.027
	LMSS-NAS	0.901 ± 0.027	80.2 ± 6.8	0.123 ± 0.034	14/18	0.633± 0.031	51.8 ± 1.9	0.273 ± 0.020
	BNN-NAS	0.859 ± 0.022	84.4 ± 5.2	0.238 ± 0.036	14/18	0.556± 0.027	41.9 ± 3.2	0.395 ± 0.030
	PEP-NAS	0.893 ± 0.038	85.4 ± 6.1	0.176 ± 0.044	14/18	0.658± 0.018	55.3 ± 2.6	0.289 ± 0.024
	This Work	**0.930** ± **0.015**	**88.5** ± **5.1**	**0.108** ± **0.032**	**17/18**	**0.672**± **0.021**	**60.7** ± **1.8**	0.237 ± 0.021

CHB-MIT, Children’s Hospital Boston Massachusetts Institute of Technology Scalp EEG Database; AUC, area under the curve; FPR, false-positive rate; STS-HGCN, spatio-temporal-spectral hierarchical graph convolutional network ; ViT, Vision Transformer; HViT, hybrid vision transformer; SALT, Long Short-Term Memory-Transformer and Self-Supervised Learning; SL-NAS, efficient and lightweight convolutional neural network architecture search; LMSS-NAS, Lightweight Multiscale neural architecture search with Spectral-Spatial attention; BNN-NAS, Neural Architecture Search for Binary Neural Networks; PEP-NAS, Progressive Evaluation and sub-Population preservation Neural Architecture Search

**Table 4.** Experimental results on the AES dataset. All experiments are implemented under identical experimental settings, and each experiment is repeated more than twice. The optimal value of each evaluation metric is marked in bold.

Architecture design	Method	Patient-specific				Patient-independent		
		AUC	*S_n_* /%	FPR	*P* < 0.05	AUC	*S_n_* /%	FPR
Artificial design	STS-HGCN	0.842 ± 0.023	82.8 ± 5.3	0.176 ± 0.024	3/4	0.526 ± 0.020	46.9 ± 2.8	0.417 ± 0.021
	ViT	0.830 ± 0.021	81.3 ± 5.4	0.155 ± 0.031	3/4	0.509 ± 0.025	48.2 ± 3.0	0.376 ± 0.025
	HViT	0.846 ± 0.030	86.2 ± 4.7	0.125 ± 0.017	4/4	0.581 ± 0.018	59.1 ± 2.4	0.352 ± 0.024
	SALT	0.821 ± 0.025	84.2 ± 5.6	0.142 ± 0.031	4/4	0.572 ± 0.023	53.7 ± 2.1	0.438 ± 0.029
Automated design	SL-NAS	0.833 ± 0.034	86.8 ± 5.8	0.183 ± 0.028	4/4	0.557 ± 0.031	55.4 ± 2.9	0.391 ± 0.018
	LMSS-NAS	0.855 ± 0.028	82.5 ± 5.7	0.159 ± 0.020	4/4	0.594 ± 0.020	51.5 ± 3.1	0.451 ± 0.032
	BNN-NAS	0.817 ± 0.037	79.8 ± 6.3	0.201 ± 0.029	3/4	0.538 ± 0.026	49.8 ± 2.2	0.465 ± 0.024
	PEP-NAS	0.859 ± 0.039	83.7 ± 5.1	0.119 ± 0.023	4/4	**0.620** ± **0.015**	59.6 ± 2.5	0.347 ± 0.027
	This Work	**0.878** ± **0.015**	**87.3** ± **4.3**	**0.107** ± **0.015**	**4/4**	0.617 ± 0.017	**61.3** ± **2.2**	**0.339** ± **0.024**

AES, American Epilepsy Society Seizure Prediction Challenge Dataset; AUC, area under the curve; FPR, false-positive rate; STS-HGCN, spatio-temporal-spectral hierarchical graph convolutional network ; ViT, Vision Transformer; HViT, hybrid vision transformer; SALT, Long Short-Term Memory-Transformer and Self-Supervised Learning; SL-NAS, efficient and lightweight convolutional neural network architecture search; LMSS-NAS, Lightweight Multiscale neural architecture search with Spectral-Spatial attention; BNN-NAS, Neural Architecture Search for Binary Neural Networks; PEP-NAS, Progressive Evaluation and sub-Population preservation Neural Architecture Search

**Table 5.** Experimental results on the private dataset. All experiments are implemented under identical experimental settings, and each experiment is repeated more than twice. The optimal value of each evaluation metric is marked in bold.

Architecture design	Method	Patient-specific				Patient-independent		
		AUC	*S_n_* /%	FPR	*P* < 0.05	AUC	*S_n_* /%	FPR
Artificial design	STS-HGCN	0.825 ± 0.029	76.2 ± 5.7	0.185 ± 0.025	16/20	0.593 ± 0.029	42.1 ± 2.3	0.381 ± 0.020
	ViT	0.846 ± 0.032	81.5 ± 6.0	0.169 ± 0.018	17/20	0.608 ± 0.025	39.5 ± 3.0	0.329 ± 0.031
	HViT	0.902 ± 0.022	83.1 ± 4.1	0.152 ± 0.026	18/20	0.637 ± 0.026	47.8 ± 1.9	0.276 ± 0.030
	SALT	0.883 ± 0.027	79.6 ± 5.5	0.167 ± 0.032	16/20	0.625 ± 0.031	45.5 ± 2.2	0.295 ± 0.025
Automated design	SL-NAS	0.871 ± 0.025	82.8 ± 5.5	0.159 ± 0.022	17/20	0.619 ± 0.022	44.9 ± 2.8	0.344 ± 0.025
	LMSS-NAS	0.817 ± 0.036	77.4 ± 4.3	0.198 ± 0.029	16/20	0.584 ± 0.028	44.7 ± 1.9	0.392 ± 0.022
	BNN-NAS	0.859 ± 0.038	78.9 ± 6.1	0.217 ± 0.033	16/20	0.579 ± 0.024	46.3 ± 2.4	0.357 ± 0.029
	PEP-NAS	0.894 ± 0.023	81.8 ± 4.4	0.154 ± 0.025	18/20	0.651 ± 0.019	49.4 ± 2.7	0.308 ± 0.026
(lr)2–9	This Work	**0.923** ± **0.015**	**84.4** ± **4.0**	**0.138** ± **0.021**	**19/20**	**0.662** ± **0.020**	**51.6** ± **2.4**	**0.253** ± **0.019**

AUC, area under the curve; FPR, false-positive rate; STS-HGCN, spatio-temporal-spectral hierarchical graph convolutional network ; ViT, Vision Transformer; HViT, hybrid vision transformer; SALT, Long Short-Term Memory-Transformer and Self-Supervised Learning; SL-NAS, efficient and lightweight convolutional neural network architecture search; LMSS-NAS, Lightweight Multiscale neural architecture search with Spectral-Spatial attention; BNN-NAS, Neural Architecture Search for Binary Neural Networks; PEP-NAS, Progressive Evaluation and sub-Population preservation Neural Architecture Search

#### Results on the CHB-MIT dataset

Under the patient-specific evaluation paradigm, CMS-NAS achieves competitive overall performance and outperforms all compared baseline methods in this work. It obtains an AUC of 0.930, which is 0.008 higher than HViT (0.922), the best-performing manually designed model, and 0.037 higher than PEP-NAS (0.893), the top NAS baseline. The sensitivity reaches 88.5%, which is higher than that of all compared methods, verifying the model’s strong ability to predict epileptic signals. The FPR is as low as 0.108, which effectively reduces the probability of false alarms in clinical practice. According to statistical tests, 17 out of 18 subjects meet P<0.05, demonstrating that its performance superiority is statistically significant. Further analysis shows that manually designed networks rely on fixed topologies and manual prior knowledge, which limits their flexibility to adapt to interpatient variations in EEG features. Models such as ViT and STS-HGCN are constrained by a single feature extraction paradigm and cannot simultaneously capture local time-frequency details and global temporal variations of EEG signals. Among the compared NAS methods, the models only adopt general search spaces. Most of them are merely optimized based on CNN, and no task-specific customization is conducted for epileptic EEG tasks, leading to notable deficiencies in feature mining capability. Combining the hybrid architecture of CNN, Mamba, and SNN with a dedicated search space tailored for EEG tasks, CMS-NAS can adaptively extract discriminative features from the interictal and preictal periods of individual patients. For this reason, it outperforms all baseline models in both recognition accuracy and false alarm control.

Under the more challenging patient-independent paradigm, all models suffer from performance degradation to varying degrees, while CMS-NAS remains in the leading position. It achieves an AUC of 0.672 and a sensitivity of 60.7%, outperforming all manually designed models and conventional NAS models on these 2 core metrics, with only a slightly lower FPR than HViT. The results reveal that the inherent limitations of fixed network architectures and the general search strategies of existing NAS methods are further exacerbated in cross-patient scenarios, making it difficult to extract shared epileptic pathological features across different subjects. Through architecture search, CMS-NAS realizes complementary advantages among the 3 network types: CNN captures universal local time-frequency features, Mamba models long-range temporal dependencies, and SNN leverages the sparse spike mechanism. Ultimately, the proposed method delivers stronger feature generalization capability for cross-patient tasks.

#### Results on the AES dataset

In the patient-specific scenario, CMS-NAS achieves better performance across all metrics than the compared baselines, with an AUC of 0.878, a sensitivity of 87.3%, and an FPR of merely 0.107. All 4 experimental samples satisfy P<0.05, indicating strong statistical significance. In the patient-independent cross-subject evaluation, CMS-NAS achieves the highest sensitivity of 61.3% among all methods. Its AUC is 0.617, only marginally lower than PEP-NAS (0.620), and the FPR also outperforms other baseline algorithms. Overall, PEP-NAS obtains a slight advantage in AUC with its general search architecture, but it is inferior to our method in sensitivity and FPR. When processing canine intracranial data, which differs substantially from human scalp EEG in acquisition protocols and data distribution, CMS-NAS still maintains excellent performance. This demonstrates that the proposed framework possesses strong cross-data adaptability and remarkably higher robustness compared with competing algorithms.

#### Results on the private dataset

In the patient-specific scenario on the clinical private dataset, CMS-NAS achieves an AUC of 0.923, a sensitivity of 84.4%, and an FPR of 0.138, surpassing all manually designed models and NAS baselines on all 3 core metrics. Among 20 clinical patients, 19 pass the significance test with P<0.05. For the patient-independent cross-patient clinical generalization task, CMS-NAS achieves the highest values across all metrics among the compared methods, with an AUC of 0.662, a sensitivity of 51.6%, and an FPR of 0.253. Evaluated on the private dataset that closely resembles real clinical scenarios, CMS-NAS achieves favorable cross-patient generalization performance among all compared methods and presents great potential for practical clinical applications.

Comprehensive cross-dataset comparisons lead to the following conclusions: Manually customized networks rely on domain prior knowledge to acquire decent performance for single-patient prediction but are restricted by empirical limitations and fail to extract universal cross-patient features effectively; canonical NAS approaches eliminate manual design bias and deliver better generalization than hand-designed networks, yet their architecture search lacks task-oriented constraints tailored for EEG analysis, restricting the upper bound of fine-grained single-subject identification; and the proposed CMS-NAS integrates domain prior information and automated architecture optimization, achieving comprehensive performance gains in both individual-specific modeling and cross-patient generalization tasks.

### Comparison of deployment performance

To verify the lightweight property and real-time inference performance of the proposed CMS-NAS, comprehensive comparative analyses between manually designed architectures and automatically searched NAS-based architectures are conducted from 5 perspectives: architecture search cost, parameter quantity, model storage size, multiply-accumulate operations (MACs), and inference latency. All experiments were carried out on a single NVIDIA RTX 4090 graphics card under identical experimental configurations. The quantitative results are listed in Table 6. Manual design approaches require no architecture searching time yet suffer unbalanced optimization and drastically varied structural redundancy. Such drawbacks are particularly prominent in STS-HGCN and SALT. Manually tailored models fail to balance model size and inference efficiency, and these drawbacks hinder their practical deployment on edge devices.

**Table 6.** Comparison of deployment performance. Bold values show the optimal results of deployment performance in each column.

Architecture design	Method	Search time /h	Params /M	Size /MB	MACs /M	Latency /ms
Artificial design	STS-HGCN	-	0.341	1.300	119.383	19.469 ± 0.820
	ViT	-	0.137	0.523	1.931	1.603 ± 0.031
	HViT	-	0.137	0.523	46.430	5.103 ± 0.076
	SALT	-	7.538	28.755	255.544	20.603 ± 0.216
Automated design	SL-NAS	20	0.940	3.585	131.349	27.722 ± 0.293
	LMSS-NAS	15	0.053	0.204	24.597	12.149 ± 0.080
	BNN-NAS	**7**	0.557	2.125	6.589	2.666 ± 0.089
	PEP-NAS	12	0.076	0.290	54.511	13.515 ± 0.058
	This work	11	**0.034**	**0.131**	**0.293**	**1.308** ± **0.019**

MACS, multiply-accumulate operation; STS-HGCN, spatio-temporal-spectral hierarchical graph convolutional network ; ViT, Vision Transformer; HViT, hybrid vision transformer; SALT, Long Short-Term Memory-Transformer and Self-Supervised Learning; SL-NAS, efficient and lightweight convolutional neural network architecture search; LMSS-NAS, Lightweight Multiscale neural architecture search with Spectral-Spatial attention; BNN-NAS, Neural Architecture Search for Binary Neural Networks; PEP-NAS, Progressive Evaluation and sub-Population preservation Neural Architecture Search

Although traditional automated NAS methods are capable of optimizing network architectures automatically, they generally suffer from high search cost and unbalanced performance optimization. In contrast, the proposed CMS-NAS constructs a lightweight and efficient search space tailored for epileptic seizure prediction. It adopts a multiobjective evolutionary algorithm together with a multiobjective weighted scoring scheme to evaluate and optimize network architectures, thereby achieving the favorable trade-off of overall performance. The entire search process takes only 11 h, delivering moderately superior search efficiency. Meanwhile, the model maintains a lightweight level in terms of parameter count and storage size. Its MACs reach as low as 0.293 M, which is the minimum among all compared methods, and the inference latency is merely 1.308 ms, well satisfying the requirements of real-time prediction. These results fully validate the effectiveness of CMS-NAS in balancing prediction accuracy and model size for epileptic seizure prediction.

### Ablation experiments

#### Ablation experiments of multiobjective weight parameters

To validate the capability of CMS-NAS in boosting prediction accuracy and reducing model parameters during architecture search, all experiments start from a fixed initial population by configuring distinct values of $\lambda_{\mathrm{acc}}$ and $\lambda_{\text{size}}$ within the multiobjective scoring function. The average $F1_{\text{loss}}$ and model size of all individuals in the population are recorded throughout evolutionary iterations. As illustrated in Fig. 5, the optimization prioritizes the objective assigned with a larger weight and converges faster toward the Pareto frontier. Moreover, the Pareto distribution of the final-generation population reveals that setting both $\lambda_{\mathrm{acc}}$ and $\lambda_{\text{size}}$ to 1 achieves favorable trade-off between the 2 optimization objectives. These observations prove the overall efficacy of CMS-NAS for simultaneous accuracy improvement and parameter reduction.

**Fig. 5.**
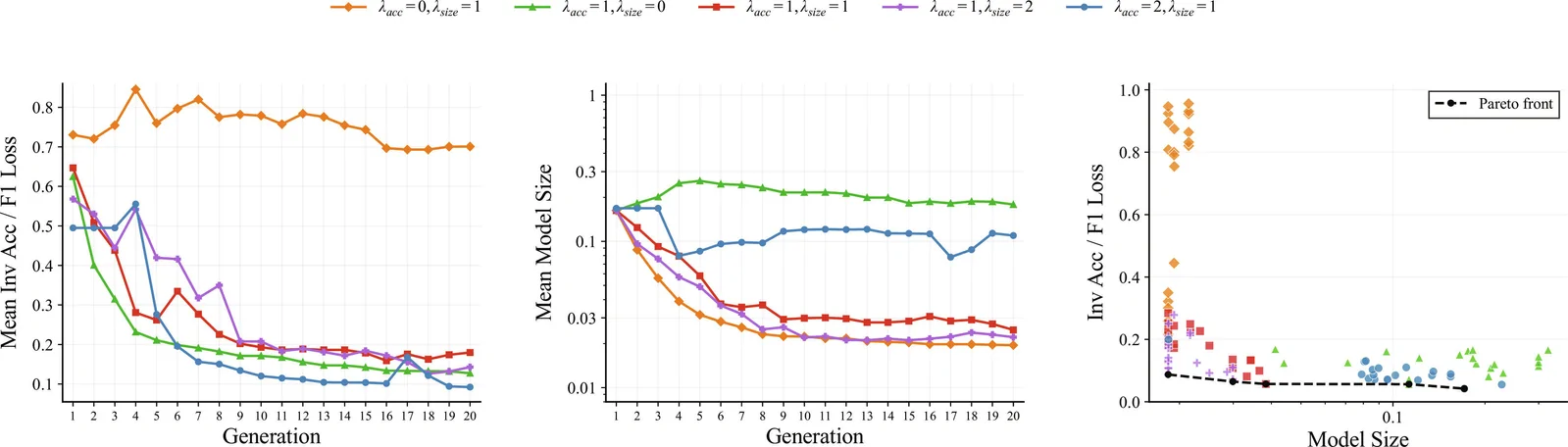
The figure intuitively illustrates the average prediction accuracy and average parameter size of all individuals in each generation during population evolution, as well as the distribution of individuals belonging to the final evolved population.

#### Statistical analysis on selection frequency of basic modules

We further statistically analyze the selection frequency of the 2 basic modules in the NAS search process. As plotted in Fig. 6, the proportion of the 2 modules remains relatively stable during searching, with slight discrepancies under varied weight configurations. Specifically, the algorithm prefers the MambaSpike module when $\lambda_{\mathrm{acc}}$ dominates, whereas the ConvSpike module is favored under a larger $\lambda_{\text{size}}$. Such results demonstrate complementary advantages of the 2 modules in prediction accuracy and parameter consumption.

**Fig. 6.**
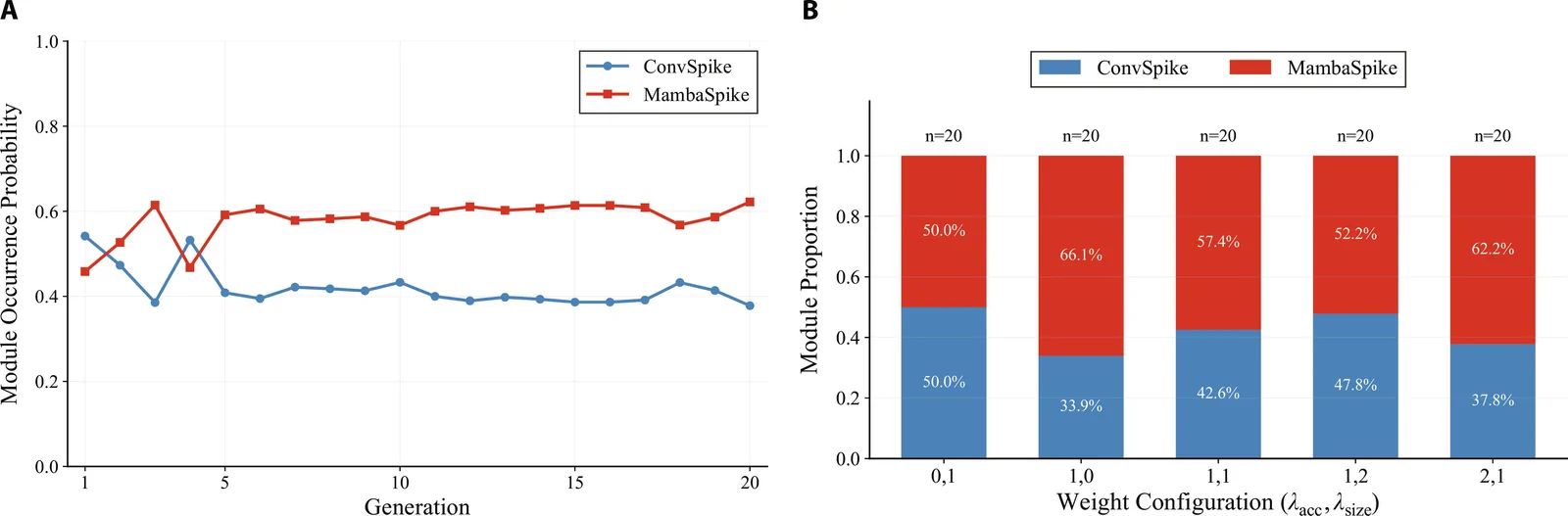
Panel (A) shows the usage proportion of the 2 types of block throughout the entire architecture search process, while panel (B) illustrates their proportions in the final generation under different multiobjective weighting coefficients.

#### Ablation experiments for search space

To further verify the individual contribution of core modules in CMS-NAS, ablation tests are implemented on CNN, Mamba, and SNN. A fixed initial population is adopted for the whole searching pipeline, and both $\lambda_{\mathrm{acc}}$ and $\lambda_{\text{size}}$ are set to 1. The quantitative outcomes listed in Table 7 indicate that each core component contributes positively to overall model performance via single-component and dual-component ablation validations.

**Table 7.** Ablation experiments for search space. Bold values represent the optimal results for the metric in each column on three datasets, respectively.

Dataset	Method			Patient-specific				Patient-independent		
	CNN	Mamba	SNN	AUC	*S_n_* /%	FPR	*P* < 0.05	AUC	*S_n_* /%	FPR
CHB-MIT	×	✓	✓	0.925	**89.2**	0.124	17/18	0.658	58.3	0.249
	✓	×	✓	0.923	85.7	0.121	16/18	0.661	57.6	0.258
	✓	✓	×	0.917	86.3	0.116	17/18	0.655	56.2	0.245
	✓	×	×	0.907	84.5	0.133	16/18	0.632	54.1	0.274
	×	✓	×	0.921	85.3	0.127	16/18	0.647	57.0	0.263
	×	×	✓	0.893	84.2	0.142	15/18	0.631	55.8	0.296
	✓	✓	✓	**0.930**	88.5	**0.108**	**17/18**	**0.672**	**60.7**	**0.237**
AES	×	✓	✓	0.876	86.1	**0.102**	4/4	0.604	59.2	0.348
	✓	×	✓	0.862	85.6	0.125	4/4	0.591	57.5	0.356
	✓	✓	×	0.871	86.7	0.118	4/4	0.598	60.4	0.351
	✓	×	×	0.847	84.2	0.149	4/4	0.586	56.3	0.372
	×	✓	×	0.865	84.9	0.132	4/4	0.594	57.9	0.353
	×	×	✓	0.843	83.5	0.158	3/4	0.579	58.6	0.381
	✓	✓	✓	**0.878**	**87.3**	0.107	**4/4**	**0.617**	**61.3**	**0.339**
Private	×	✓	✓	0.921	83.2	0.185	19/20	0.642	48.4	0.281
	✓	×	✓	0.911	81.0	0.177	17/20	0.613	47.9	0.316
	✓	✓	×	0.919	84.4	0.149	19/20	0.651	49.5	0.270
	✓	×	×	0.904	79.2	0.188	17/20	0.619	45.2	0.331
	×	✓	×	0.909	82.1	0.162	18/20	0.637	48.6	0.298
	×	×	✓	0.904	79.2	0.188	17/20	0.619	45.2	0.331
	✓	✓	✓	**0.923**	**84.4**	**0.138**	**19/20**	**0.662**	**51.6**	**0.253**

CNN, convolutional neural network; SNN, spiking neural network; CHB-MIT, Children’s Hospital Boston Massachusetts Institute of Technology Scalp EEG Database; AES, American Epilepsy Society Seizure Prediction Challenge Dataset; AUC, area under the curve; FPR, false-positive rate

According to the results of dual-module combination experiments based on single-component removal, all 3 core modules play important and complementary roles in the overall performance. The absence of any component will lead to degradation of the model’s overall performance to varying degrees. For groups with only 1 module retained, the model suffers an even sharper performance decline, which indicates that a single network architecture is difficult to fully satisfy the complex requirements of seizure prediction.

Combining all ablation results, we can draw a clear conclusion: CNN, Mamba, and SNN are not simply stacked together but form an organic integration with complementary functions and collaborative working mechanisms. CNN is dedicated to extracting local time-frequency features and realizing lightweight parameter control. Mamba excels at global long-range temporal modeling and cross-population generalization. SNN undertakes sparse denoising and enhancement of transient abnormal signals. These 3 modules respectively address the 3 major challenges of EEG signals, namely, nonstationarity, long-term temporal dependency, and high noise. The above results fully verify the excellent rationality and applicability of the custom hybrid search space designed specifically for epileptic seizure prediction in this work.

## Discussion

This study targets the EEG-based epileptic seizure prediction task and proposes CMS-NAS, a hybrid architecture search framework integrating CNN, Mamba, and SNN. Centered on a multiobjective evolutionary algorithm, the framework realizes automatic iterative optimization of network structures. It completes population initialization via custom encoding rules and adopts a population update strategy combining elite preservation, crossover, and mutation. This mechanism not only inherits excellent architectures to guarantee steady performance improvement but also maintains population diversity to prevent the search process from falling into local optima.

As shown in Fig. 5 and 6 and Table 7, a series of ablation experiments further validate the effectiveness of each module and optimization strategy. Component ablation on the search space reveals that removing any core module leads to decreased AUC and sensitivity as well as increased false FPR, demonstrating that the 3 types of networks perform distinct and indispensable functions. In addition, different weight combinations tend to bias the algorithm toward a single optimization objective. When both the accuracy weight $\lambda_{\mathrm{acc}}$ and the size weight $\lambda_{\text{size}}$ are set to 1, the population can stably converge to the Pareto optimal front, achieving the favorable trade-off between prediction performance and model lightweight design. Further statistical analysis on the selection frequency of basic modules shows that the algorithm prefers the MambaSpike module when prioritizing accuracy, whereas the ConvSpike module is selected more frequently for lightweight requirements. The 2 hybrid modules can be dynamically combined according to practical demands, which further enhances the adaptability of the network.

As shown in Tables 3 to 6, the proposed method exhibits great advantages in both seizure prediction performance and deployment performance. Comparative experiments on the CHB-MIT, AES, and private clinical datasets demonstrate that CMS-NAS outperforms manually designed models and NAS baselines across core metrics including AUC, sensitivity and FPR, in both patient-specific individualized prediction and the more challenging patient-independent cross-population generalization tasks. Furthermore, we evaluate the practical deployment capability of the model from 5 perspectives: architecture search time, parameter quantity, model storage size, MACs, and inference latency. Experimental results show that the total search time of CMS-NAS is only 11 h, which achieves high search quality with acceptable time cost. The finally searched model has merely 0.034 M parameters, a storage size of 0.131 MB, MACs of 0.293 M, and an inference latency of 1.308 ms. It outperforms all compared methods in all deployment-related metrics involved in this experiment, highlighting the advantages of the proposed framework in lightweight deployment. Furthermore, since relevant studies on NAS in the field of seizure prediction are currently scarce, all compared NAS methods adopted in this paper are recent representative works migrated from other research fields.

Although the proposed method achieves satisfactory performance in prediction accuracy, module effectiveness, and deployment efficiency and addresses the core drawbacks of traditional NAS models including excessive parameters and high computational complexity, as well as eliminating the reliance on expert experience required by manually designed networks, several limitations still exist in this study. Currently, our method is designed for fixed datasets and specific tasks and only adopts single-modal EEG signals. A high-quality NAS system is expected to support multimodal epilepsy prediction. Therefore, future work will explore integrating multimodal epilepsy prediction into the NAS framework and enabling the searched networks to adapt to diverse tasks, so as to further validate the universality and practicality of the proposed framework.

## Conclusion

This paper proposes an end-to-end DL framework named CMS-NAS based on multiobjective evolutionary algorithm for seizure prediction. The proposed CMS-NAS simultaneously optimizes prediction accuracy and parameter quantity on 3 experimental datasets. Sufficient comparative experiments are conducted against various baseline methods under identical experimental settings across all 3 datasets. CMS-NAS achieves competitive performance in both patient-specific and patient-independent evaluation protocols, and the automatically searched network architectures bring lower deployment cost in practical applications. Moreover, comprehensive ablation studies are performed to verify the effectiveness of each constituent component within CMS-NAS.

In conclusion, the presented method remedies the prominent drawbacks including excessive structural redundancy of handcrafted networks, expensive searching cost, and heavy computational overhead of conventional NAS algorithms. It achieves a favorable trade-off among search efficiency, model lightweight property, and real-time inference, which makes CMS-NAS more suitable for practical clinical deployment of EEG-based seizure monitoring.

## Data Availability

The data and materials used to support the findings of this study are available from the corresponding author upon reasonable request.
